# Antimicrobial drugs for persistent diarrhoea of unknown or non-specific cause in children under six in low and middle income countries: systematic review of randomized controlled trials

**DOI:** 10.1186/1471-2334-9-24

**Published:** 2009-03-03

**Authors:** Katharine Abba, Rebecca Sinfield, C Anthony Hart, Paul Garner

**Affiliations:** 1 Liverpool School of Tropical Medicine, Pembroke Place, Liverpool, L3 5QA, UK

## Abstract

**Background:**

A high proportion of children with persistent diarrhoea in middle and low income countries die. The best treatment is not clear. We conducted a systematic review to evaluate the effectiveness of antimicrobial drug treatment for persistent diarrhoea of unknown or non-specific cause.

**Methods:**

We included randomized comparisons of antimicrobial drugs for the treatment of persistent diarrhoea of unknown or non-specific cause in children under the age of six years in low and middle income countries. We searched the electronic databases MEDLINE, EMBASE, LILACS, WEB OF SCIENCE, and the Cochrane Central Register of Controlled Trials (CENTRAL) to May 2008 for relevant randomized or quasi randomized controlled trials. We summarised the characteristics of the eligible trials, assessed their quality using standard criteria, and extracted relevant outcomes data. Where appropriate, we combined the results of different trials.

**Results:**

Three trials from South East Asia and one from Guatemala were included, all were small, and three had adequate allocation concealment. Two were in patients with diarrhoea of unknown cause, and two were in patients in whom known bacterial or parasitological causes of diarrhoea had been excluded. No difference was demonstrated for oral gentamicin compared with placebo (presence of diarrhoea at 6 or 7 days; 2 trials, n = 151); and for metronidazole compared with placebo (presence of diarrhoea at 3, 5 and 7 days; 1 trial, n = 99). In one small trial, sulphamethoxazole-trimethoprim appeared better than placebo in relation to diarrhoea at seven days and total stool volume (n = 55).

**Conclusion:**

There is little evidence as to whether or not antimicrobials help treat persistent diarrhoea in young children in low and middle income countries.

## Background

In 2002, diarrhoea caused an estimated 13.2% of child deaths worldwide[1], most of them in children under the age of five years in low and middle income countries[2]. In this group, around 3% to 19% of acute diarrhoea episodes become persistent[3] and some experts estimate that up to 50% of diarrhoea deaths may be due to persistent diarrhoea[1]. As the number of deaths from acute diarrhoea reduces following widespread use of oral rehydration therapy, the contribution of persistent diarrhoea to overall diarrhoea mortality is increasing. Persistent diarrhoea also adversely affects nutritional status, and is often associated with malnutrition. In one study, three months after a persistent diarrhoea episode, children had significantly lower weight for age and weight for height Z scores than three months before the episode[4].

Children living in poor areas with poor hygiene and sanitation conditions and children with poor nutritional status are most at risk of developing persistent diarrhoea[3]. As poor nutrition is both a risk factor and a consequence of persistent diarrhoea, the two are very commonly associated. Children with HIV/AIDS are at particular risk; at initial presentation to hospital with HIV/AIDS, around 36–50% [5-7] of children have persistent diarrhoea. Dysentery and more severe diarrhoeal illness are more likely to become persistent than milder episodes[3]. Previous antibiotic use and irrational use of antibiotics for acute diarrhoea are also considered to be risk factors[3] for persistent diarrhoea.

### Definition and causes

The World Health Organization (WHO) defines diarrhoea as the passing of three or more loose stools (which take the shape of a container) within a 24 hour period. A new episode of diarrhoea can occur after two full days without diarrhoea. Episodes of diarrhoea lasting for less than 14 days are defined as acute, episodes lasting for 14 or more days are defined as persistent.

The causes of persistent diarrhoea in populations are complex and poorly understood, and in individuals are often unknown. Pathogens associated with persistent diarrhoea are also often found in healthy children without diarrhoea[4], a finding highlighted in our related review of pathogens found in the stool of children with persistent diarrhoea and without diarrhoea, to be published at a later date. Some, such as *Cryptosporidium*, *Giardia lamblia *and enteroaggregative *Escherichia coli *(EAggEC) are thought to be associated with persistent diarrhoea[3] in some locations. Children with persistent diarrhoea who are HIV positive may have different patterns of enteric pathogens than those who are HIV negative[8]. In addition, the diarrhoea may be caused by multiple factors: malnutrition, micronutrient deficiencies, milk or food intolerance, or diseases of the bowel, as well as prior antimicrobial therapy[9].

### Treatment

The current recommendations of Integrated Management of Childhood Illness programme[10] for treating diarrhoea is that children with bloody diarrhoea are treated with antimicrobials for *Shigella*, or for *Entamoeba histolytica *where the organism is detected in the stool; it is recommended that children with watery diarrhoea are not treated with antimicrobials; except where *Giardia lamblia *is found.

Even where an enteric pathogen is detected in children with persistent diarrhoea, it is not always clear that this is the cause of the illness. In addition, health workers in low and middle income countries often do not have access to sufficient high quality diagnostic laboratory facilities to analyse stool samples for all children with diarrhoea. In these situations treatment needs to be syndromic, based on symptoms and the mostly likely cause of the symptoms; and may include replacement fluid and electrolytes, nutritional rehabilitation and sometimes drug treatment[9].

Recent studies have suggested that antimicrobial treatment may be useful in causes of watery diarrhoea other than *Giardia lamblia*. For example, there have been promising trials looking at the use of nitazoxanide for children with diarrhoea associated with *Cryptosporidium *infection[11], and ciprofloxacin for diarrhoea associated with enteroaggregative *E.coli *in adults with AIDS[12]. However, these are yet to be demonstrated in a systematic review or large scale trial in children with persistent diarrhoea. The use of antimicrobials needs to be approached with caution due to potential problems of drug resistance and possible reactions of some micro-organisms: entero-haemorrhagic *E coli *(EHEC) may release toxins more readily when a person is treated with certain drugs, potentially causing severe illness[13].

Given the lack of diagnostic facilities, and the consequent requirement that children presenting to health facilities with persistent diarrhoea receive only presumptive treatment, we conducted a systematic review. This project was originally requested by WHO, due to lack of evidence in this area.

### Objective

To assess the effectiveness of antimicrobial drugs for the treatment of persistent diarrhoea of unknown or non-specific aetiology in children under the age of six years in low and middle income countries. Only children under the age of six were included, because this age group are most at risk from death or serious morbidity relating to persistent diarrhoea.

### Criteria for including studies in this review

#### Types of studies

Randomized and quasi-randomized controlled trials.

#### Types of participants

Children under the age of six years, with diarrhoea of unknown or non-specific aetiology longer than 14 days duration, in a low or middle income country setting (as defined by the World Bank). Trials including only participants with a known cause for their diarrhoea were excluded. Trials including people of different ages or with diarrhoea of different durations were included if data relating only children under the age of six with persistent diarrhoea could be extracted.

#### Types of intervention

Intervention: any antimicrobial drug treatment regimen plus usual care

Control: placebo or usual care

#### Types of outcome

Primary:

Duration of diarrhoea

Secondary:

Presence of diarrhoea at follow-up

Need for hospitalisation

Stool volume

Death

Adverse events:

Any adverse events

### Search strategy for identification of studies

The search strategy was developed in collaboration with an information retrieval specialist (see below). The strategy listed applies to Medline and was amended where necessary to search the other databases listed. No language restrictions were applied. The reference lists of included studies were also scrutinised for additional relevant studies. The last search was undertaken May 20 2008.

### Search Strategy

#### Databases searched

MEDLINE (1966 to March 2007) via the OVID interface (table 1 for strategy).

**Table 1:** Reasons for excluding reports initially identified as relevant

*Reason for exclusion*	*Number of reports*
Did not assess the use of antimicrobials	14

Included only patients with a specific cause of diarrhoea	6

Used an alternative definition of persistent diarrhoea	3

Included adults only	3

Included children with acute diarrhoea only	3

Were review articles	3

Had no control group or were case studies	3

Were undertaken in a high income country	1

Relevant controlled trial but no mention of randomization	1

EMBASE (1980 to March 2007) via the OVID interface.

LILACS database – Latin American and Caribbean Health Sciences Literature (1982 to date) – via Virtual Health Library interface.

WEB OF SCIENCE (Science Citation Index Expanded – 1945 to present).

Cochrane Central Register of Controlled Trials (CENTRAL), published in *The Cochrane Library*.

#### Search strategy in MEDLINE

1. persistent (diarrhea OR diarrhoea) ti, ab.

2. chronic (diarrhea OR diarrhoea) ti, ab.

3. watery (diarrhea OR diarrhoea) ti, ab

4. (diarrheal disease*) OR (diarrhoeal disease*) ti, ab

6. 1 OR 2 OR 3 OR 4) NOT cancer NOT (inflammatory bowel disease*) NOT (ulcerative colitis)

7. child* OR infant* OR pediatr* ti, ab

8. 6 AND 7

9. Diarrhea, infantile/drug therapy [MeSH] OR Diarrhea, infantile/prevention and control [MeSH] OR Diarrhea, infantile/therapy [MeSH]

10. Diarrhea/drug therapy [MeSH] OR Diarrhea/prevention and control [MeSH] OR Diarrhea/therapy [MeSH]

11. therap* OR treatment OR treating ti, ab

12. 9 OR 10 OR 11

13. Anti-Infective Agents/administration and dosage [MeSH] OR Anti-Infective Agents/adverse effects [MeSH] OR Anti-Infective Agents/therapeutic use [MeSH])

14. Antiprotozoal Agents/administration and dosage [MeSH] OR Antiprotozoal Agents/adverse effects [MeSH] OR Antiprotozoal Agents/therapeutic use [MeSH])

15. Antiparasitic Agents/administration and dosage [MeSH] OR Antiparasitic Agents/adverse effects [MeSH] OR Antiparasitic Agents/therapeutic use [MeSH])

16. 12 OR 13 OR 14 OR 15

17. 8 AND 16

18. randomized controlled trial [pt] OR controlled clinical trial [pt] OR randomized controlled trials [MeSH] OR controlled clinical trials [MeSH] OR random allocation [MeSH] OR double-blind method [MeSH] OR single-blind method [MeSH]

OR (placebos [MeSH] OR placebo* ti, ab OR random* ti, ab OR quasi-random* ti, ab

19. 17 AND 18

## Methods

### Study selection

Two reviewers independently inspected titles and abstracts identified in the initial literature search in order to identify potentially relevant publications. All potentially relevant publications identified by at least one reviewer were obtained in full text format. One reviewer then applied the inclusion criteria to select which trials to include in the review, and scrutinised publications for duplication of trial results.

### Assessment of methodological quality

Two reviewers independently assessed the methodological quality of the included trials, using a pro-forma as a guide. The methodological quality of the included trials was assessed in terms of generation of the allocation sequence and allocation concealment and reported as adequate, inadequate or unclear according to Juni 2001[14]. We recorded who was blinded in each trial. We classified inclusion of randomized participants in the analysis as adequate if 80% or more of the participants are included in the analysis, unclear if not described, and inadequate if less than 80% were included. Any disagreements were resolved by discussion.

### Characteristics of included trials

One reviewer summarised the characteristics of the included trials. Information was extracted on the trial setting, location, start date, participant characteristics (such as age, sex and nutritional status), number of participants, intervention, control and outcomes for each trial. We also noted any acknowledged sources of financial support for the trial.

### Data extraction

One author extracted outcomes data for the intervention and control groups. For dichotomous data we extracted the number of participants with the outcome, the total number randomized to each group, and the total number included in the analysis. For continuous data we extracted the number of participants in each group, the arithmetic mean and their standard deviations, where available.

### Data analysis

The analysis was undertaken using RevMan 4.2 software. For dichotomous data we calculated relative risks and where appropriate combined results from different trials. Where continuous data were summarized by arithmetic means, we summarized the results using weighted mean difference (WMD). We stratified the analysis by class of antimicrobial drug used. We presented data on adverse events in a narrative summary.

## Results

### Studies identified

Four trials met our inclusion criteria. The initial search identified 378 publications, from which we selected 43 that appeared, from their abstracts or titles, to be potentially relevant, for retrieval of the full text. We were unable to assess three reports due to time constraints, one because it was not available within the UK, and two, published in Polish and Ukrainian respectively, because translators were not available within the available time period. Of the 40 papers that we were able to assess, three were reports of trials eligible for inclusion in the review. The reasons for the exclusion of the other 37 are summarised in Table 1. We identified one additional eligible trial through reading the reference lists of retrieved review articles.

### Characteristics of included trials

The characteristics of the four included trials are summarised in Table 2, and also described below.

**Table 2:** Characteristics of Included Trials

Location and date of publication	Participants	Setting	Interventions	Outcomes	Source of Support
Guatemala 1992 [18]	Number: 102 Girls and boys, 3–35 mths Diarrhoea 14–18 days, weight for length not < -2 Z, no dysentery	Surveillance project: rural indigenous community Field nurse visited homes three times daily to deliver intervention	Group 1: 10 mg oral gentamicin sulphate per kg body weight per day: 3× daily for 5 days. Group 2: Placebo: 1% magnesium sulphate 3× daily for 5 days.	Diarrhoea stopped at 7 days, and at least 48 hrs after end of treatment	None stated

India 1992 [15]	Number: 68 Boys, 3 mths to 4 yrs Diarrhoea ≥ 14 days, weight for length ≤ 90% of standard, no dysentery	Hospital oral rehydration unit in New Delhi Field worker visited homes twice daily to deliver intervention and leave evening dose	Group 1: 50 mg oral gentamicin per kg body weight per day: 4 times daily for 6 days Group 2: Placebo	≤ 2 liquid stools per day at 6 days. Intake of IV fluids, ORS, water and energy. Output of diarrhoea, vomit and urine. Weight change at 168 hrs	Diarrhoeal Diseases Control Programme, World Health Organization

India 1996 [16]	Number: 156 Girls and boys, 4 mths to 3 yrs Diarrhoea ≥ 14 days <4 weeks, no dysentery, *Giardia lamblia *or *Entamoeba histolytica*, no illness requiring antibiotics	Surveillance project with referrals to clinic and direct clinic attendances: urban slum in Delhi (outpatients)	Group 1: 30 mg oral metronidazole plus 50 mg oral nalidixic acid per kg body weight: 3× daily for 7 days Group 2: 30 mg oral metronidazole per kg body weight: 3× daily for 7 days Group 3: Placebo	First of 3 days with < 3 liquid stools in a 24 hrs; days 3, 5 and 7. No. stools in previous 24 hrs; days 2, 5 and 7. Weight change at days 7 and 14	CDR, World Health Organization

Bangladesh 1995 [17]	Number: 55 Girls and boys, 6 to 15 mths Diarrhoea ≥ 14 days <6 weeks No systemic infection, antibacterial use previous 7 days, severe malnutrition, *Vibrio cholerae, Salmonella, Shigella, Giardia lamblia *or *Entamoeba histolytica*	Clinical research unit of specialist diarrhoea hospital (inpatients)	Group 1: 10 mg oral trimethoprim plus 50 mg sulphamethoxazole per kg body weight; 2× daily for 7 days Group 2: Placebo,	Diarrhoea stopped at 7 days. Stool output (g/kg) days 1,2,3,4,5,6 and 7, plus all days 1–7 combined. Duration of diarrhoea. Hospital infection. Energy intake (kcal/kg/day)	United States Agency for International Development and the International Centre for Diarrhoeal Disease Research, Bangladesh

### Location

Three trials were undertaken in the South East Asia region; two in India[15,16] and one in Bangladesh[17]. Another was undertaken in Guatemala [18].

### Dates of fieldwork

Two trials recruited participants during the period 1988 to 1990[15,18]. Two trial reports did not provide dates for the fieldwork; these were published in 1995[17] and 1996[16] respectively.

### Participants

Each trial included children of a slightly different age range, the total age range being three months to four years. Three trials excluded children with diarrhoea lasting over a certain length of time (18 days[18], 4 weeks[16] and 6 weeks[17] respectively). Three trials excluded children with dysentery or with blood in the stool[15,16,18], and the other[17] excluded children with *Shigella *or *Entamoeba histolytica *(the main causes of dysentery). One trial excluded children described as 'severely malnourished', another stipulated that weight for length was not less than -2 Z [17,18], while another included only children who had weight for length less than or equal to 90% of the standard [15]. Two trials excluded children with systemic infection[16,17], and one excluded children who had taken antibiotics in the previous seven days[17]. Two excluded children with specific enteric pathogens (*Giardia lamblia *and *Entamoeba histolytica *in one[16], *Giardia lamblia*, *Entamoeba histolytica, Vibrio cholerae, Salmonella and Shigella *another[17]). None included specifically children with HIV/AIDS.

### Settings

Participants were recruited from a range of sources including a diarrhoea surveillance project within a rural indigenous community[18], referrals to a hospital rehydration unit[15], and attendance at a clinic provided as part of a surveillance project in an urban slum[16]. In one trial it was not clear how the participants were recruited[17]

### Comparisons

Two trials compared gentamicin with placebo[15,18], one three-arm trial compared metronidazole, metronidazole combined with nalidixic acid, and placebo[16], and one trial compared sulphamethoxazole-trimethoprim with placebo[17]. All antimicrobials were given orally for between five and seven days.

### Outcomes

Only one trial reported on our primary outcome of duration of diarrhoea[17]. All four trials reported on recovery from diarrhoea by the end of treatment; one trial also assessed diarrhoea at three and five days[16]. Three trials reported on stool output at various time points; two measured stool weight[15,17], and one measured number of stools[16]. In addition, two trials reported on weight gain at different time intervals[15,16], one reported on various fluid intakes and outputs[15], two reported on energy intake[15,17], and one reported on hospital-acquired infections[17]. Two trials recorded and reported on adverse events[15,18].

### Sources of support

Two trials were supported by the World Health Organization[15,16], one by the United States Agency for International Development and International Centre for Diarrhoeal Disease Research[17], and one did not mention a source of support[18]. None acknowledged support from pharmaceutical companies.

### Quality assessment

A summary of the methodological quality assessment for each study is presented in Table 3. All four studies described blinding the participants to which group they were in, and including over 80% of the randomized participants in the main analyses. Three studies also reported adequate generation of allocation sequence, allocation concealment and blinding of service providers and outcomes assessors. The remaining study[16] was unclear on these methodological issues, but stated that it used randomization, and that it used coded antimicrobial and placebo preparations, which were identical in appearance.

**Table 3:** Methodological quality assessment of included trials

*Location and reference*	*Generation of allocation sequence*	*Concealment of allocation*	*Blinding*			*Percentage of participants included in the analysis*
				
			*Participants*	*Providers*	*Outcomes assessors*	
Guatemala [17]	Adequate	Adequate	Yes	Yes	Yes	Adequate

India [14]	Adequate	Adequate	Yes	Yes	Yes	Adequate

India [15]	Unclear	Unclear	Yes	Unclear	Unclear	Adequate

Bangladesh [16]	Adequate	Adequate	Yes	Yes	Yes	Adequate

### Outcomes

#### Oral gentamicin versus placebo

Two trials[15,18] compared oral gentamicin (10 mg/kg body weight in one trial and 50 mg/kg body weight in the other) versus placebo. Both trials assessed and reported on presence of diarrhoea at end of treatment (6 or 7 days). Combining the results of the two trials, there was no difference between the gentamicin and placebo groups in the number of children with diarrhoea at the end of treatment (relative risk 1.04, 95% CI 0.78 to 1.38, 151 participants); around half the children in both groups recovered within six or seven days. There was no difference in point estimates between the two trials, one of which excluded children with weight for length less than -2 Z and used a standard dose of gentamicin [18], (RR = 0.01), and one of which included only children with weight for length equal to or less than 90% of the standard and used a massive dose of gentamicin with the aim of eradicating aerobic bacterial overgrowth of the small intestine [15] (RR = 0.10). There were also no significant differences between groups in any reported measure of weight gain, fluid intake, energy intake, or fluid output.

One trial reported no drug-related untoward effects[18]. The other reported no clinical toxicity, and blood urea concentrations similar in the treatment and placebo groups[15].

#### Metronidazole combined with nalidixic acid versus metronidazole alone versus placebo alone

One trial[16] compared three treatment groups: metronidazole combined with nalidixic acid, metronidazole alone and placebo. There was no significant difference between the groups in the primary outcome of time to recovery, or in the number of children with diarrhoea at three, five or seven days, although point estimates tended to favour the group receiving metronidazole combined with nalidixic acid.

There were no significant differences between groups in the mean number of stools in the previous 24 hours at three days or five days; at seven days the group receiving metronidazole combined with nalidixic acid had fewer stools than the group receiving metronidazole alone but the difference was small (mean difference -1.10, 95% confidence interval -2.07 to -0.13, 99 participants), and not significantly different when compared with the placebo group. There were no differences between the groups in percentage weight gain at seven and 14 days.

Adverse events were not mentioned in the report of this trial.

#### Sulphamethoxazole-trimethoprim versus placebo

One trial compared sulphamethoxazole-trimethoprim with placebo[17]. Significantly fewer children in the treatment group had diarrhoea at the end of treatment (7 days) (relative risk 0.4, 95% confidence interval 0.16 to 0.99, 55 participants). This related to an 82% cure rate in the antimicrobials group and 55% cure rate in the placebo group. Duration of diarrhoea also appeared to favour treatment, but the difference was not significant. Total stool output in the seven days following start of treatment was significantly lower in the treatment group (mean difference -179.4 g, 95% confidence interval -340.20 to -18.60).

Participants in the treatment group had a significantly lower risk of acquiring infections while in hospital compared with placebo (relative risk 0.06, 95% confidence interval 0.01 to 0.54); while energy intake from the hospital diet was similar in both groups.

Adverse events were not mentioned in the report of this trial.

## Discussion

Despite the comprehensive search strategy used, we identified only four trials assessing the use of antimicrobials for children with persistent diarrhoea in low and middle income countries; all of which were conducted in the late 1980s and early 1990s. All four trials used good quality methods to minimise the risk of bias. However, they are also all quite small, involving between 55 and 156 participants; and so the effect estimates are consequently imprecise. Two trials excluded children with certain laboratory-confirmed pathogens known to cause diarrhoea found in the stool, while the other two trials did not test the participants for specific pathogens. Two trials included only children with associated malnutrition or low weight for height. None of the studies included children with known HIV/AIDS.

In a related review that has been submitted for publication, we examined the frequency of different pathogens found in children with persistent diarrhoea and no diarrhoea. We found that, while children with persistent diarrhoea are more likely to have at least one detectable enteric pathogen than children without diarrhoea, for specific pathogens there were no significant differences between the two groups; both exhibit a wide range of different pathogens in the stool, including bacteria, parasites and viruses. These findings suggest that antimicrobial therapy may be of limited effectiveness in the majority of children with persistent diarrhoea. Trials of oral gentamicin, a non-absorbable drug effective against a wide range of bacteria, have tended to confirm this. Small trials of sulphamethoxazole-trimethoprim, and metronidazole combined with nalidixic acid have suggested some potentially worthwhile effects, which have not been tested in larger trials. Both these combinations are absorbable and effective against a wide range of bacteria; possible modes of action therefore include direct action on the enteric pathogens, and also on any systemic infections which may be delaying the child's recovery from diarrhoea.

## Conclusion

There is limited evidence as to whether or not antimicrobials help to reduce the duration of persistent diarrhoea or reduce its health impact in young children in developing countries, either in children with a symptomatic diagnosis of persistent diarrhoea where no laboratory exists, or in children with persistent diarrhoea in whom known bacterial and parasitic causes have been excluded. There is currently insufficient data to recommend the use of any kind of antibiotic in persistent diarrhoea of unknown cause or non-specific cause, and hence no implications for current guidelines on the treatment of persistent diarrhoea.

Further good quality trials, of sufficient sample size to detect clinically important effect sizes, are needed to evaluate the use of antimicrobials in the presumptive treatment of persistent diarrhoea. These trials should be conducted in areas where persistent diarrhoea is common in young children, and where testing for enteric pathogens is not routinely available. Children with HIV/AIDS should be included. Trials should first assess the treatment combinations that have already given encouraging results in previous, smaller trials, and then perhaps other wide-spectrum antimicrobial drug combinations. Outcomes should include nutritional recovery, which will require a longer period of follow-up than that of the trials included in this review. Monitoring of emerging pathogen resistance should be undertaken within these trials.

## Competing interests

The authors declare that they have no competing interests.

## Authors' contributions

KA wrote the review protocol, inspected the initial search results for potentially relevant publications, selected studies for inclusion, extracted data, undertook the data analysis and drafted the manuscript. RS inspected the initial search results for potentially relevant publications, extracted data, and assisted in the interpretation of data. CAH provided technical advice and assisted in the interpretation of the findings. PG conceived of the study, secured its funding, participated in the design, assisted with the interpretation of data and helped to draft the manuscript. PG, KA and RS read and approved the final manuscript. CAH died before the completion of the manuscript. He was a Professor within the Department of Infections and Host Defence, School of Medicine, University of Liverpool.

## Pre-publication history

The pre-publication history for this paper can be accessed here:

http://www.biomedcentral.com/1471-2334/9/24/prepub
